# Exploring Customer Journeys in the Context of Dentistry: A Case Study

**DOI:** 10.3390/dj11030075

**Published:** 2023-03-07

**Authors:** Bhaven Modha

**Affiliations:** Whittington Health NHS Trust, Hillingdon Community Dental Service, Uxbridge Dental Centre, 1 Redford Way, Uxbridge UB8 1SZ, UK; b.modha@nhs.net

**Keywords:** dentistry, patients, patient care, health behaviour, marketing of health services, advertising, consumer behaviour, digital technology, dental practice management

## Abstract

This case study aims to explore how customer journey concepts can apply to a hypothetical scenario, centring on a patient (customer persona) within the dentistry arena, and with a particular focus on special care dentistry. As an educational exercise, this paper may inform dental and allied professionals on how aspects of the customer journey notion may be embedded into their own practices, so that patient-centricity might be better optimised. The hypothetical scenario considers the organisational context, customer persona, contemporary customer purchase decision-making models, and marketing approaches. These components are used to create a customer journey map to help visualise and identify the varying customer–business interactions. The customer journey, focussing on the awareness, initial consideration, active evaluation, pre-purchase, purchase and post-purchase stages, is then conceptually analysed. The analyses reveal that there are areas of friction, attributable to numerous factors. The case study recommends that by introducing digitalisation and omnichannel marketing, alongside existing internally generated and multi-channel marketing approaches, considerable improvements may be achievable. As the patient technology landscape becomes more digital and dental organisations face fiercer competition, dental care providers relying on traditional marketing approaches may well need to adapt and introduce innovative, yet cost-effective digitalisation and omnichannel marketing approaches. Nevertheless, dental care providers, and dental and allied professionals must uphold an underlying duty of care, ensuring that all practises are legal, decent, honest, truthful, and above all ethical.

## 1. Introduction

The World Health Organization advocates that “the right to health for all people means that everyone should have access to the health services they need, when and where they need them, without suffering financial hardship” [1]. Healthcare can, therefore, be seen as a fundamental human right. However, the economics of delivering healthcare will be a major consideration for many service providers, who will wish to provide high-quality patient care at the lowest possible cost [2]. Therefore, healthcare can also be seen as a business.

Patients are people whose “status is greatly reduced by illness or injury that renders them vulnerable, frightened, often in pain, medicated, exhausted and confused” [3], while customers are “people who enjoy elevated status by virtue of their potential to purchase goods or services” [3]. It has been suggested that the term “patient” is best used in a paternalistic model of healthcare that involves identifying disease and managing it [4,5]. Patients shall often need to make important yet challenging decisions about their treatment options (that is, “goods” or “products” in the business context). Hoping to attain improved health, patients will then select (that is, “purchase” in the business context) such options. Many patients shall want their clinicians’ care (that is, “service” in the business context) to be grounded in altruism and professionalism, as opposed to receiving a typical type of “customer service”, which may not be genuine. Thus, patients are not ordinary customers, and perceiving them as so can be a grey area [6,7,8]. Nonetheless, some scholars recommend regarding patients as customers [9,10,11].

Customers remain essential to the profitability of businesses, and establishing long-term relationships with them will be imperative, and customers will need to have strong, positive, worthwhile experiences or “customer journeys”. A customer journey is defined as a “customer’s interactions with one or more service providers to achieve a specific goal” [12]. It involves customers’ cognitive, affective and sensory evaluations of a chain of interactions with businesses’ service delivery systems [13,14]. Most businesses will want their customers’ journeys to be fulfilling, so that they can be continually repeated and thus, continually profitable. This may be achieved by maximising customer-centricity, which involves recognising and providing high-quality and value to individual customers, instead of mass or target markets [15].

Businesses can investigate whether they are customer-centric by creating a fictional portrayal of an ideal customer or “customer persona”; this can be based on the evaluation of market research and/or actual data about existing customers [16]. This customer persona will have several interactions with a business at varying moments in time. Such interactions and experiences may determine whether a potential customer becomes an actual customer, and whether an actual customer is retained as a loyal customer. Therefore, one’s customer journey can be a significant tool for analysis. Relatedly, it has been asserted that within healthcare, the quality of a patient’s experience affects satisfaction, loyalty behaviours, clinical effectiveness, overall wellbeing, and quality of life [13].

This case study explores how the aforementioned concepts may apply to a hypothetical scenario, which focuses on an individual dental patient (customer persona) within the dentistry arena, in a special care dentistry setting. The customer journey is conceptually analysed, and is used to guide recommendations on how the patient’s experience may be enhanced by incorporating aspects of digital transformation. As an educational exercise, this may inform dental and allied professionals on how aspects of the customer journey notion can be integrated into their own practices, so that patient-centricity might be better optimised.

## 2. Organisational Context

In the United Kingdom, most dental care services are provided by the government-funded National Health Service (NHS) body. NHS dental services are provided in primary (i.e., general dental practice), secondary (i.e., specialist care in hospitals, universities or regional centres) and tertiary (i.e., specific specialist care in specialist hospitals, universities or regional centres) care settings. Most dental patients are first seen within primary care. Should a patient require a more complex procedure, beyond the capabilities of a primary care general dental practitioner (GDP), they can subsequently be referred onwards. Secondary care is responsible for serving patients that cannot be seen within primary care (i.e., specialist orthodontics, oral surgery, endodontics, and periodontics, amongst others), whilst tertiary care is responsible for treating patients that cannot be treated in secondary care (i.e., dental treatment requiring cross-disciplinary and multi-professional teams) [17].

NHS England provides independent contracts to dental practices and corporate bodies, allowing primary, secondary and tertiary dental care services to be delivered. Patients receiving NHS dental care within general dental practice and community dental settings are required to pay a contribution to the cost of dental services, which are laid out by NHS England. However, those patients in receipt of certain state benefits or exemptions may be free from paying for NHS dental costs. Patients of secondary and tertiary NHS dental care receive their treatment cost-free [17].

From the outset, patients have a choice as to whether they visit an NHS, private or mixed NHS–private dental practice that delivers primary care services. The key differences between NHS and private dentistry [18,19] have been outlined (Table 1). It is unknown whether private dental care is superior to its NHS counterpart [20].

In the hypothetical scenario, the organisation of interest is a dental practice, located in an English borough. More specifically, this is a community dental practice, the only one within the borough, treating patients with special care needs. As a type of secondary care, exclusively NHS service, it is responsible for providing comprehensive dental care to patients with disabling conditions that cannot be treated in primary care. The types of disabling conditions can include: dental phobia, complex medical conditions, severe mental health problems including dementia, and moderate to severe learning disabilities, amongst others. Within the borough, there also exists one private practice, which offers dental services to patients with only mild disabling conditions.

The NHS special care dental practice accepts patient referrals from GDPs, and health and social care professionals, and such patients must be based within the specific borough. On average, 1500 referrals are received each year. Referrals are triaged by administrative and clinical staff to assess their acceptability against eligibility criteria; referrals failing to meet these are returned to the referrer. Those referrals, which are deemed eligible, are placed on a waiting list which on average is six months. Because the service faces a great demand than they are funded to supply, patients failing to confirm or attend an appointment without any prior communication or extenuating circumstance are discharged from the service.

The NHS special care dental practice has approximately 3250 patients that are dependent on its dental services. If a patient is deemed capable of receiving the same level of care within primary dental care, then such patient is discharged to the referring GDP or is offered information on accessing other primary care dental services within the borough.

## 3. The Customer Persona

By formulating a customer persona that is from a customer’s perspective, businesses can better examine and understand the circumstances that transpire in their customers’ lives, which consequently, directs them to purchase a product or service [21]. Therefore, a customer persona must encapsulate the key elements of a customer’s needs and experiences [22]. As part of the hypothetical scenario, a fictional patient (customer persona) has been created (Table 2).

## 4. Contemporary Customer Purchase Decision-Making Models

Customer purchase decision-making refers to the process where customers make important decisions about the products and services they purchase, the quantity they purchase, and the time, place and means of purchase. This also involves customers, first, thinking about where best to spend their valuable resources: time, money, and efforts; second, customers choosing an option out of a few or many; and third, customers selecting an alternative option out of a few or many [23].

Playing a pivotal role in decision-making processes, customer behaviour is defined as “the process and activities people engage in when searching for, selecting, purchasing, using, evaluating, and disposing of products and services so as to satisfy their needs and desires” [24]. Customer behaviour and decision-making processes can be strongly influenced by factors including: demographics, economics, marketing mix, situational circumstances, perceptions, attitudes, motivation, personality, learning, family, appurtenance group, reference group, social class, culture and subculture [25].

Many customer purchase decision-making models exist within the literature. However, one that features prominently comprises five sequential stages: (i) recognition of need or problem; (ii) information search; (iii) comparing the alternatives; (iv) purchase; (v) post-purchase evaluation [26]. By building upon this classic model and adapting it accordingly, many scholars have introduced new models to further understand the customer journey. Of these, the buyer decision-making model [27] contains five similar stages: (i) need or problem recognition; (ii) information search; (iii) evaluation of alternatives; (iv) purchase decision; (v) post-purchase behaviour. However, it further considers information acquisition and evaluation behaviours, as these can contribute towards a purchase decision and influence post-purchase behaviour.

Another contemporary model, the McKinsey customer decision journey model [28], employs a cyclical route. It encompasses five stages: (i) initial consideration; (ii) active evaluation; (iii) moment of purchase; (iv) post-purchase experience; (v) loyalty loop. Within this, customers select one brand out of many, and this eventually leads them to a final destination of loyalty. Here, customers are absorbed into a “loyalty loop”, transforming them into ongoing customers that can bypass the preliminary initial consideration and active evaluation stages.

## 5. Marketing Approaches

Marketing is defined as “the activity, set of institutions, and processes for creating, communicating, delivering, and exchanging offerings that have value for customers, clients, partners, and society at large” [29], whilst advertising is defined as “the placement of announcements and messages in time or space by business firms, non-profit organizations, government agencies, and individuals who seek to inform and/or persuade members of a particular target market or audience regarding their products, services, organizations or ideas” [30].

Internally generated marketing involves “building a customer-oriented culture in an organization with the purpose to increase awareness among the customers and employees and ensure a prosperous co-creation of value” [31]. Examples include a practice brochure, business cards, in-house information centres such as a notice board or glass enclosed case, thank you cards, and direct mail [32]. It is thought that internally generated marketing can heighten employees’ enthusiasm, dedication and job satisfaction, which subsequently increases the quality of service [33].

Multi-channel marketing involves strategic interactions with potential customers on varying platforms or channels [34]. These include: a website (i.e., the dental practice’s website); a retail location (i.e., the dental practice’s premises); a promotional event (i.e., staff making oral health promotion visits to local community centres); or word-of-mouth (i.e., from patients, and their family or caregivers; staff, referrers, and other relevant stakeholders).

Omnichannel marketing involves strategic interactions with potential customers via several multi-channels, which might provide them with a more integrated experience [34]. Examples include: mobile channels such as smart phones, tablets, applications and social media; and mass communication channels such as television, radio and print; amongst others.

One contemporary approach for employing efficient omnichannel marketing via a fully digital approach has been demonstrated [35]. Named the “RACE” model, its four stages include: (i) “reach” the correct audience; (ii) “act” by ensuring prompt interactions with potential customers; (iii) “convert” by transforming these potential customers into actual customers by offering excellent services; (iv) “engage” by promoting repeat business, customer loyalty and advocacy. This methodology can also incorporate an initial phase of “plan”; this involves generating the business’ overall digital strategy and objective setting [35].

## 6. The Customer Journey Map

With the customer persona in mind, the typical interactions between the customer and the business can be illustrated by mapping the customer’s journey, entirely from the customer’s perspective [16]. Customer journey maps are a well-recognised method of analysis to help strengthen customer experiences within commercial business settings. They are regarded as highly necessary in developing insight and empathy with customers [36]. Such maps typically take the form of flow-type diagrams that depict, with realism, the customer’s experiences, points of view, and emotional responses across time [37,38].

**Figure 1:**
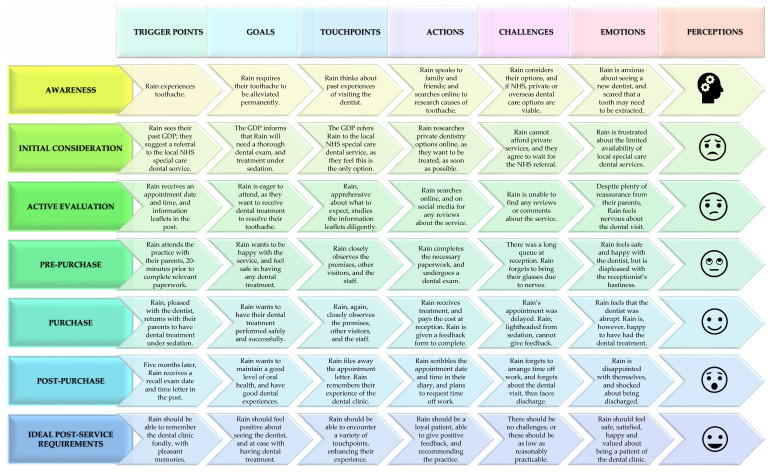
Customer journey map.

By using the hypothetical scenario’s customer persona, together with amalgamating and adapting aspects of the aforementioned customer purchase decision-making and McKinsey customer decision journey models, a customer journey map can be formulated (Figure 1), and conceptual analyses can be made.

### 6.1. Awareness Stage

Dental pain is a major trigger, which can lead a patient to contemplate the need to see a dentist [39]. Rain first realises that they need dental care because of their toothache. Rain has several options. They can attend the dental practice, which they visited five years ago, but they are reluctant due to having some negative, off-putting experiences there. Rain could make arrangements to visit the university-based dental practice, but this is far away and impractical. Rain could research alternate options (i.e., other dental practices) and seek recommendations from friends and family. Through this, a neighbour recommends dental treatment abroad, as this may be cheaper. Rain is afraid to pursue this option due to possible postoperative complications.

Rain’s own research via searching online and/or by phoning and visiting various dental practices will be productive, yet requires time and energy. Through this, Rain learns that private dental care is costly and unaffordable, owing to their personal circumstances. Due to a scarcity of options, Rain resorts to arranging an appointment with the NHS dental practice that they visited during childhood. They are familiar with its setup, and they do not want to spend any more valuable time performing research.

### 6.2. Initial Consideration Stage

Touchpoints are defined as “distinct points in the experience of contacts between the company and the customer”, and they include “cognitive, emotional, behavioral, sensorial, and social components” [40]. Thus, touchpoints are crucial for reaching customers, captivating and drawing them close to a business. An initial key touchpoint, encountered by Rain is the GDP. Rain’s dental need (that is, “consumer need” in the business context) is to have their toothache eliminated. Due to Rain’s dental phobia and anxiety, the GDP recommends a referral to the borough’s NHS special care dental service. The GDP feels that Rain will only cope with dental treatment under sedation or general anaesthesia. Because the GDP provides no alternative options, Rain feels obliged to agree to the referral.

### 6.3. Active Evaluation Stage

Rain encounters a further touchpoint when receiving their appointment and information leaflets in the post. Wanting their dental problem to be addressed, as soon as possible, Rain feels that they must accept the allocated appointment.

Rain may wish to learn further about the NHS special care dental practice via its website and by searching for reviews on reputable websites. Due to its specialised dental service and unique groups of patients, Rain may not have friends or family members that are patients of this dental practice. Moreover, the dental practice does not have any online presence beyond a simple and stationary website. Without online reviews, recommendations, or opinions from friends or family, some patients may experience increased anxiety about their impending dental visits.

### 6.4. Pre-Purchase Stage

An initial consultation is first required, so that the dentist can examine Rain and determine the applicable treatment options. For Rain to be seen for a dental exam at the NHS special care dental practice, Rain must first agree to have a dental exam [17]. Rain is not entitled to any benefits or exemptions and therefore, must pay for NHS dental costs; they feel that these are affordable compared to private costs.

Rain, accompanied by their parents, attends their appointment 20-min earlier to complete the relevant registration and medical history forms. Rain is very nervous and forgets to wear their spectacles; thus, they take longer to complete the paperwork. There is a long queue at the reception, and Rain is unimpressed with feeling hurried by the receptionist.

Later, Rain has a very positive experience with the dentist, who they thought was empathetic and kind. This experience encourages Rain to consent to the proposed dental treatment. Rain and their parents are therefore, enthused to book a treatment appointment at the reception.

### 6.5. Purchase Stage

Accompanied by their parents, Rain attends for their dental treatment. This is delayed, as the dentist is overrunning, and the waiting adds to Rain’s nerves. Rain is eventually seen and successfully treated; Rain and their parents are very happy with this achievement. However, they are slightly disheartened that the dentist gave quick and abrupt verbal postoperative instructions. Rain understands that this may be due to time pressures that the dentist may have experienced. Nonetheless, Rain is relieved that the treatment is complete, and their toothache should now resolve. Rain is therefore, happy to pay the cost of the NHS dental treatment.

### 6.6. Post-Purchase Stage

Rain cannot concentrate on completing a feedback questionnaire at the reception due to side effects of the sedation; they ask their parents to complete this on their behalf. Rain is relieved to not have to attend the dental practice until another six months. As their dental pain has been alleviated, Rain can fully focus on their career and professional goals.

When Rain receives their six-month recall exam appointment date and time in the post, they note this down in their calendar. However, overcome with work pressures, Rain forgets about their dental appointment, failing to confirm and attend it. Consequently, Rain receives a letter in the post, notifying them that they are discharged from the NHS special care dental service. Rain is disappointed with this and at the prospect of having to invest time and efforts into being re-referred. Resultantly, Rain is not engaged in a “loyalty loop”.

## 7. Discussion

### 7.1. Customer (Patient) Journeys in Private Dental Practice

The hypothetical scenario’s dental practice provides a much needed special care dental service, which is ultimately a governmentally funded NHS service. It is well publicised that NHS dentistry is chronically underfunded [41], whilst private dentistry is thriving [42]; this may give rise to inequalities and differing experiences amongst NHS and private patients.

Private dental services are privately funded and thus, have a for-profit business model, where they aim to gain more money than what they spend. Contrasting from the demand-outweighs-supply service of the NHS-only special care dental practice, private dental practices may not have enough demand to fulfil their available capacity. Thus, this could be reflected in the costlier prices that private patients are required to pay.

Those patients with disabling conditions that want quicker dental care, can afford costlier private fees, and are able to travel afar may completely bypass NHS special care dental services. Since NHS services are outstretched, such patients obtaining private treatment elsewhere are unlikely to cause NHS services any significant financial constraint. However, certain private dental practices offering special care dental services may need to implement initiatives (i.e., omnichannel marketing approaches), to help them gain a competitive advantage over other similar private dental practices.

Relatedly, private dental practices may need to make much larger marketing efforts that incur high expenses. For instance, it has been reported that some private dental organisations receive marketing help from “coaching gurus” [43]. One large marketing effort, the use of celebrity endorsements in cosmetic dentistry, has been shown to influence dental patients’ buying behaviour [44].

The use of high-end technology and software, and varying omnichannel marketing approaches may offer private dental patients more streamlined customer (patient) journeys [9]. For instance, offering private dental patients’ consultations with treatment coordinators may help to make these patients feel that their individual needs are being catered to [45]. Private dental practices may also offer patients regular or flexible payment plans, and these schemes might “encourage patients to opt for new or higher cost treatments which they previously might not have considered due to the price” [46]. Such approaches may be essential for defying competition and in engaging patients in “loyalty loops”. However, private dental practice has been described as having a “commercialized context”, which might only serve to “superficially empower” dental patients [47].

### 7.2. The Special Care Dentistry Sector

The ultimate nature of NHS special care dental services is to provide high-quality and clinically necessary dental care for its unique patient groups, rather than solely driving profit. Such services face a demand that outweighs supply, as well as underfunding. These could be reasons why the hypothetical scenario’s NHS special care dental practice relies on economical and traditional internally generating, and multi-channel marketing approaches, rather than modern omnichannel marketing that may incur large start-up and maintenance costs, as well as an increased workload for staff.

Such economical and traditional approaches might lead to a lesser degree of engagement with patients and caregivers, and perhaps this might inadvertently contribute towards many special care dental patients utilising the special care dental service on a when-needed basis. Particularly, it has been emphasised that special care dental patients’ have an “ad hoc” nature of dental attendance [48]. Because of this, there is a risk that the most vulnerable of special care patients may fall out of the dental care system. This poses immense danger; especially as special care patient groups are at a greater risk of developing dental disease [49].

With a gap of six months or so until the next dental appointment, some patients may become occupied with the demands or priorities in their life. During this time, NHS special care dental services could utilise some “reach” approaches to retain their patients’ and patients’ caregivers’ interest. Maintaining an online presence with regular posts; sharing relevant news; holding quizzes and competitions; raising awareness of upcoming oral health promotion events; and appropriate blogs and podcasts on websites [50] may maintain patients’ and caregivers’ attention, perhaps acting as a reminder for any future dental appointment. Realistic communications depicting appropriate preventive messages and imagery of the consequences of dental neglect may further stress the importance of dental attendance and dental care.

Touchpoints including contact with the receptionist; notices on the waiting room display boards; literature sent in the post; social media, websites, email, SMS text message, and phone calls; amongst others, should clearly warn patients and caregivers about the consequences of not attending appointments. Providing patients with samples of toothpaste, toothbrushes, and other oral hygiene cleaning aids, as well as selling actual dental cleaning products at the dental practice’s reception at cost-effective prices, will be beneficial. For instance, instead of attending a pharmacy or supermarket, some patients may especially attend the dental practice to make such purchases.

In comparison to traditional marketing approaches, social media marketing is believed to be interactive, effective, and economical in advertising products and services, because more customers are spending time online [51]. Thus, a strong online presence through the availability of profiles on prominent social media platforms may be beneficial for NHS special care dental services. Posts on such platforms may include virtual tours of the dental clinics; images of the specialist facilities; information profiles on its staff members; pertinent oral health messages tailored to varying patient groups; blogs about service updates; and perhaps feedback and inspirational stories from patients that have been successfully treated. Mass communication channels could even be employed: a television or radio appearance on a local news network to highlight the specialist dental services. Similarly, articles in local newspapers may be beneficial [32].

With its use growing in popularity within private dentistry, teledentistry “is a combination of telecommunications and dentistry, involving the exchange of clinical information and images over remote distances for dental consultation and treatment planning” [52]. Some patients may have trouble waiting a long time for their dental appointment, and those in severe pain could resort to seeking urgent attention at an emergency dental care, out-of-hours, or dental school facility, or by even resorting to “do-it-yourself” dentistry. As a quality improvement initiative, NHS dental services could introduce a type of teledentistry service. Through this, they could triage referred patients via remote online consultations during their period of waiting. Provisional diagnoses, pain relief advice, oral health instruction, and indications of possible treatment need may help to reassure potential patients, as well as build rapport. A similar approach has been used by speech and language therapists for children with cerebral palsy [53].

While special care dental patients are often considered to be an underrepresented group, the number of patients requiring special care dentistry is increasing and is expected to increase significantly. In particular, older people are living longer with more complex medical conditions; the prevalence of dementia is growing; increasing numbers of children with complex disabilities are surviving into adulthood; and an increasing number of older people are developing disabilities. Many of these cohorts are perceived as being marginalised in today’s society [54]. Great efforts, thus need to be made in understanding, engaging with, and being inclusive to such patient groups, and their families and caregivers.

Moreover, on both a public and professional level, the special care dentistry sector could perhaps benefit from greater recognition and inclusion within the dental industry, so that the care of special care patients is integrated, as much as possible, into mainstream primary care services; special care patients are able to receive equitable care, and the needs of the most vulnerable in society are not overlooked.

Relatedly, GDPs will need to ascertain whether patients truly have dental phobia and/or anxiety, and they will need to make best efforts to treat such patients within primary care, utilising appropriate behavioural management approaches. If such efforts are unsuccessful, then GDPs may wish to consider referrals to secondary care special care dental services, and if appropriate, perhaps psychological services including cognitive behavioural therapy. It has been suggested that GDPs require increased guidance and information about available care pathways and that patients should be given information on accessing psychological services in the management of their dental anxiety [55], to ensure a holistic care approach.

### 7.3. Removing Digital Exclusion

It has been stated that more than 12 million persons within the UK do not have basic digital skills. This mainly includes those persons that are vulnerable to social exclusion: 60% do not have qualifications; 57% are above 65-years of age; and 49% are disabled. Recent data highlight that approximately two-thirds of persons aged 75 and a third of 65–74-year-olds do not use the internet, compared to 17% of 55–64-year-olds and 5% of people aged below 55-years. Furthermore, a high “drop-out rate” of internet usage has been reported amongst older populations [56]. Attributable factors can include: disability, social isolation, cost, a lack of skills and knowledge of the internet, perceiving that the internet is not beneficial to them, and a worry that the internet might replace social interactions [57].

To reduce inequalities and to promote equality amongst all patient groups, so that each can receive a similar range of care and experience similar customer (patient) journeys, significant and sustained efforts will need to be made. Sophisticated strategies, professional support, and encouragement may be required. Particularly, suitable technologies and support ought to be accessible for persons with disabilities, cognitive impairments, and other special needs. It has been suggested that health coaches, public health nurses, administrative staff, and community volunteers may be able to support patients to use and understand various digital tools [56]. Likewise, patients’ families and caregivers who partake in decision-making should be supported accordingly.

### 7.4. Using Ethical Marketing

Due to their general convenience and ease of use, many patients are using platforms such as online social networks, groups, forums, and websites that contain other patients’ anecdotal comments; this may help patients to gather healthcare-related information and/or to make healthcare decisions [58]. Use of electronic communications: social media, websites, mobile phone applications, software, email, and SMS text messages, amongst others, may be cost-effective and could provide the shortest customer (patient) journey [59]. However, this approach does not consider the customer’s entire experience. Particularly within healthcare spheres, patients require a large level of individualisation and personalisation of their journey. Henceforth, prioritising “straight to consumer” or virtual experiences over physical ones could be deemed as ignoring an underlying duty of care [60], which could form a barrier. In particular, face-to-face interactions will be far more invaluable for welcoming special care patients and their caregivers, and in maintaining good rapports with them.

The experience a patient has with the dentist and allied professionals will be influential to whether the patient consents to any treatment and thus, whether they “convert” to actual patients. For instance, beyond possessing adept practical skills, good interpersonal communication skills have been deemed essential in marketing efforts [61]. It has also been indicated that empathy is an integral component in the dentist–patient relationship [62], and professional competence, experience, psychosocial skills to include patience, respect, and patient involvement in decision-making are also considered highly valued characteristics [63]. Thus, dental professionals, and even allied teammates to include receptionists and administrators must adhere to the principles laid out in the General Dental Council’s *Standards for the Dental Team* [64] and *Standards for Dental Professionals* [65] documents.

It has been asserted that the ever-increasing competition within the dental industry, driven by an increased usage of the internet, has caused dentistry to become more commercialised, leading to “dentist shopping” and “inappropriate, misleading, and dishonest advertising” [66]. It has also been argued that “advertising and the active “selling” of oral health services are all designed to create dental consumers, not to empower them” [47]. Not only might such ploys activate customer (patient) journeys where patients already have high expectations, leading to a potential over-promising and under-delivery of services, there is a real hazard that this could worsen an already existing perception that dentists “do not deliver a service that meets patients’ expectations and that they are driven by money” [67]. Some scholars advise the need for dental professionals to undergo education in dental marketing in order to increase their awareness of the ethical implications [68].

Relevant stakeholders must consider the adverse effects that such marketing and advertising could have on vulnerable patient groups: special needs, disabilities, facial deformities, mental health problems, of low socioeconomic status, children, adolescents, and young women, amongst others [69]. Therefore, it is vital that all dental practices, and dental and allied professionals strike a sensible and practicable balance between using traditional and up-to-date marketing vehicles that are sensitive, and consider patients’ best interests.

The problem of information asymmetry in healthcare has been longstanding. It has been recognised that “asymmetries are seen to pervade health care markets, which are characterized by high levels of uncertainty” and that “patients may be able to describe their symptoms, but they have inadequate information to relate their condition to a particular type of treatment or course of medication”; this may generate an “unequal power relationship between experts and clients which the former may exploit in their own interest” [70]. Henceforth, those working within the dental field must ensure that their marketing and advertising comply with the General Dental Council’s *Guidance on Advertising* policy [71], as well as with the Advertising Standards Authority’s regulations, which stipulate that all advertising should be “legal, decent, honest and truthful” [72].

### 7.5. Achieving Customer (Patient)-Centricity

When the mode of service delivery is patient-centred, patient value is heightened, risks are lessened, and efficiency is improved [73]. Thus, it will be important to place the patients’ interests at the heart of any developments.

Analyses of this case study’s customer (patient) journey have established that the hypothetical scenario’s dental practice utilises some traditional internally generating and multi-channel marketing approaches. At an organisational level, perhaps there may be a lack of information or motivation to implement omnichannel marketing. Underfunding within the NHS may be another contributory factor. Moreover, there may need to be a shift in the dental staff’s attitudes and behaviour, and incentives could help to achieve this [56]. Through this, staff may be more willing to embrace new ways of working, which may consequently enrich patient-centricity. It has been suggested that strategically, such issues could be managed by always thinking of customers or patients when considering digital developments [74]. In addition, dental and allied professionals have the ability to use their own personal experiences as both customers and patients, so that they can better empathise with service users.

Direct patient feedback (that is, “market research” in the business context) on the effectiveness of the current internally generating and multi-channel marketing methods, and the omnichannel marketing approaches that they might like to see in the future, should be sought from the dental practice’s patients, their family members, caregivers, and other relevant stakeholders. This could provide the dental practice with a worthwhile opportunity to implement new approaches, which could lessen any points of friction, and allow for a fusion of both traditional and contemporary vehicles. For instance, it has been highlighted that patients immensely value access to their medical records, and this can lead to increased satisfaction, as patients can save money on making transport costs and save time spent on making telephone calls [75]. In addition, by providing patients with information about their health conditions and treatment options in a digital format that is easily accessible to them, this could boost the two-way communication between the practice and its patients. Particularly, effective communication between a doctor and patient is associated with lower rates of medical errors [76].

### 7.6. Recommendations for Improving the Customer (Patient) Journey with Digitalisation

It has been affirmed that customer journeys can be strengthened by incorporating digital transformation to include digital platforms, as these may help customers and stakeholders to better connect [77]. Therefore, by combining digital transformation and omnichannel marketing, recommendations can be made to overcome areas of friction to enrich the hypothetical scenario’s customer (patient) journey (Table 3).

Although the above recommended means may have vast potential, they may not be wholly realistic or financially viable for such a governmentally funded NHS facility. As many private practices are capitalising on technological advancements and marketing innovations, NHS healthcare providers could fall behind, and this could further widen inequalities amongst differing patient groups. Thus, nurturing positive collaborative partnerships with the private sector may be beneficial for governmentally funded health services. Consequently, perhaps NHS organisations may be able to utilise private provider technological services at a reduced cost [56].

## 8. Conclusions

As dentistry continues to make advancements that improve the quality of clinical treatments, similar efforts are required to enrich overall patient (customer) journeys. As a dichotomy, dental care is also a business. Thus, dental care providers, and dental and allied professionals must reflect on the quality of their patients’ journeys, striving to enhance them. Through this, potential patients might be transformed into actual patients, and these actual patients might be transformed into loyal patients. Such a strategy may allow business to be continuously repeated, thus contributing to the business’ overall economic growth.

This case study has shown that perhaps concepts relating to customers, advertising and marketing may be applicable to patients, as long as these are in the patients’ best interests and there are altruistic intentions. The case study has also shown that customer journey mapping, a technique traditionally used within commercial business settings, can be of worthwhile use to help improve a patient’s journey, thus maximising patient-centricity within a healthcare domain. Notably, there is no single perfect customer (patient) journey or methodology, as each patient (customer) is indeed a unique person with their own decision-making processes and behaviours that can be affected by varying factors. Henceforth, great efforts are needed to become professionally well acquainted with all patients, so that each patient journey can be customised accordingly. Great endeavours are required to engage all patient groups, especially those in vulnerable and underserved sections of society; this is so that patients and associated caregivers can feel empowered, diversity can be embraced, equality and inclusivity can be fostered, and patient journeys can be satisfactorily maintained.

As the patient technology landscape becomes more digital, and the dental industry continues to become more competitive, dental care providers, utilising traditional marketing approaches, may well need to adapt and introduce innovative yet cost-effective digitalisation and omnichannel marketing. This is so that they can keep pace with their competitors in these everchanging and uncertain times. Dental care providers, and dental and allied professionals have an underlying duty of care towards their patients; thus, “straight to consumer” and virtual experiences must not completely replace physical face-to-face interactions. Ultimately, dental care providers, and dental and allied professionals must uphold an underlying duty of care and ensure that all practices within patient journeys are legal, decent, honest, truthful, and above all ethical.

## Figures and Tables

**Table 1:** Key differences between NHS and private dentistry.

	NHS Dental Services	Private Dental Services
**Scheduling of appointments**	May be less flexible due to the current shortage of NHS dentists Possible waiting lists and limited appointment slots Appointments may be of shorter duration with set timings	May be more flexible Possible availability of weekend and evening appointments Appointments may be of longer duration
**Attendance patterns**	Specific recall protocols may be in place (i.e., dental exam every six, 12 or 24 months)	Patients may be able to attend as required (i.e., dental exam or dental cleaning whenever required)
**Affordability of treatment**	Set costs determined by treatment need Set costs nationwide Varying dental treatments are categorised by a banding system Patients pay for the course of treatment that corresponds to the relevant band Patients receiving certain state benefits or exemptions may be eligible for free NHS dental care	Considerably higher costs that may vary amongst practices A fee-per-item system may be in place Dental payment plans may be available Promotional offers (i.e., free initial consultation, 20% off special offer, patient rewards, and the first ten patients will receive a free scale and polish, amongst others) may be employed
**Ethos of treatment**	Dental treatment to resolve disease, and improve form and function, without a sole cosmetic focus	Dental treatment to resolve disease and improve form and function Dental treatment that may have a sole cosmetic focus (i.e., short-term orthodontics, smile designing, and facial aesthetics, amongst others)
**Availability of specialist treatment**	May be possible within secondary and tertiary care settings but could be dependent on geographical location	May be possible within primary care private dental practices (i.e., orthodontists, endodontists, and oral surgeons, amongst others)
**Equipment, materials, and technology**	Economical usage May be restricted	A wider array may be available, which may be costlier
**Source of funding**	Government funding	Privately funded by the practice owners, and investors, amongst others

**Table 2:** Customer persona—a dental patient with special needs.

	Customer (Patient): Rain, 25-Year-Old, Newly Qualified Veterinarian
**Reason for referral**	Referred for special care dentistry by a local GDP Rain has autism but is otherwise fit and well Rain is thought to have dental phobia and anxiety Rain is too fearful of having invasive dental treatment Rain is suspected to have dental caries Rain experiences toothache that is worsening The GDP requests treatment to be carried out under sedation
**Social history**	Rain recently graduated from university Rain resides with their parents Rain has incurred a large student debt and is financially dependent on their parents Rain has secured their first job as a veterinarian and shall commence this soon Rain is currently spending quality time with friends and family
**Usage of marketing channels**	Rain has access to the internet via their mobile phone and laptop Rain actively uses social media platforms Rain reads local and national newspapers intermittently Rain watches television every day
**Motivation**	Rain is knowledgeable about science, medicine, and healthcare Rain is, however, afraid of dentists due to past negative experiences in childhood Rain has not seen a dentist in the past five years Rain’s last dental visit was at a university-based dental practice

**Table 3:** Summarised digital transformation and omnichannel marketing recommendations to enhance the customer (patient) journey.

Stage	Action
**1. Ease for customer (patient) discovery**	Effective online strategies: online searches via search engines (i.e., “dentists near me”), attaining top positions in search pages, and an increased number of patient reviews [78] Electronic patient referral system: traditional paper-based referrals could provide less timely information to specialists, in comparison to digitally sent data [79]; this could result in reduced time from the date of referral to the actual date that treatment commences
**2. Booking an appointment**	Efficient online booking system where patients can negotiate and book appointments at their own convenience; most appointment bookings occur online, outside of normal working hours [80] Clearly labelled telephone numbers that patients can obtain directly, instead of via a call centre Patients with severe and urgent issues should be able to bypass any conventional process to have an accelerated journey [81] Patients experiencing pain can be triaged and prioritised by a software system as soon as their referrals are received
**3. Before an appointment**	Implementation of teledentistry methods to ascertain and prioritise patients’ dental needs prior to their actual appointments, and to build rapport Automated communication system to confirm and welcome patients to the service Automated reminders sent to patients a week, as well as a day before their appointments Automated response service for patients to confirm attendance for their appointments Relevant forms are sent electronically to patients for completion, prior to their appointment, rather than in-house at the reception or waiting area; patients can return these via the same electronic system, since many patients tend to fill out forms prior to their initial visit [80]
**4. Arrival at the dental practice for an appointment**	Simple touchpad method at reception for patient check-ins, registrations, and submission of completed forms, enabling privacy, and eliminating possible communication barriers; most patients report that they prefer kiosks to traditional registration procedures due to reduced queuing times [82] Kiosks can incorporate a function where patients can make electronic payments, instead of making manual payments at the reception [83] Patients can utilise a contactless method: SMS text message or mobile phone application to inform the receptionist of their arrival [84]
**5. Waiting room**	Television screen on the waiting room wall, playing noteworthy oral health demonstrations, displaying important oral health messages, information about the practice, its services, policies, pricings, relevant news, upcoming oral health promotion events in the borough, and pictures of members of the dental team [85,86]; this may be informative and enhance patients’ and caregivers’ knowledge Wireless internet connectivity to enable internet usage on patients’ and caregivers’ mobile channels [87]
**6. With the dentist**	Tablet or similar device to show radiographs, video demonstrations or animations of potential dental procedures, and patient testimonials to patients and caregivers Electronic medical records can be positively impactful in terms of patient satisfaction [88]. Digital consent forms improve legibility and form completion rates, whilst decreasing errors, when compared to handwritten forms [89] Intraoral scanner use to capture direct optical impressions of patients’ teeth and mouths. This may be advantageous: reducing patient discomfort; time-efficient; simplifying clinical procedures; avoiding the need for conventional physical impressions and plaster casts; improved communication with the dental technician; and better communication and demonstration of intraoral findings with patients and caregivers [90]. Such a device might be ideal for use with certain special care patients, many of whom could have gag reflexes, phobias, and limited mouth openings, amongst others In-house camera to take before and after photographs to show patients the improvements that have been made to their teeth via the treatment; this may impress and motivate them
**7. Leaving the practice**	Patients receive an automated prompt, alerting them to traverse to the reception to complete tasks: pay any balance, book subsequent appointments, and confirm contact details, amongst others Contactless departures: contactless payment and contactless completion of documents to facilitate ease [84] Consent forms, relevant leaflets and resources, and postoperative instructions are also provided via digital means to patients: email, website link, SMS text message, or through an online portal system
**8. Feedback**	Patients are prompted to provide feedback on their experience via an electronic form: email or website link via SMS text message [78] Patients are prompted, via email or SMS text message to provide feedback on reputable websites Completing feedback at their own convenience may allow patients time to properly reflect on their experience; this may help ensure that the feedback is honest and accurate, rather than inaccurate, incomplete, rushed, or completed biasedly by an accompanying party
**9. Follow-up**	Automated sending of additional treatment information via letter, SMS text message or email [91]
**10. Returning**	Structured recall process: patients are messaged via email, SMS text message or their preferred communication channel, one month before their recall appointment, and reminder SMS text messages are sent days before the scheduled appointments; automated recalls can increase recall effectiveness [91] Highlighting positive reviews via social media and online platforms, email, or the patient’s preferred communication channel; patients are more likely to visit a dentist when the staff rating is high and when patient reviews are positive [92] Social media and online platforms, email, or the patient’s preferred communication channel could be used to highlight improvements that are being made to the special care dental services, following receipt of patients’ feedback
**11. Auditing**	Tracking progress of customer (patient) journey stages, making observations, collecting data, and reviewing the findings regularly against already established objectives Discussing real and fictional customer (patient) journeys at team meetings to highlight successes and needs for improvement, and to consider varying eventualities Utilising customer (patient) journey data to improve service delivery; thus, it is vital to identify effective patient satisfaction interventions and to investigate whether improving patient satisfaction can directly improve other important clinical outcomes [93]

## Data Availability

No new data were created or analysed in this case report. Thus, data sharing is not applicable to this case study.
